# Impact of nanotechnology at heterogeneous interphases @ Sustainability

**DOI:** 10.1016/j.heliyon.2024.e26943

**Published:** 2024-02-24

**Authors:** Pankaj Tomar

**Affiliations:** IGDTUW/GGSIPU, India

**Keywords:** Nanoscience, Carbon NPs, Biofunctionalization, Electroadhesion

## Abstract

The 21st century information and communication industries have played the pivotal role of bio-sensing technologies, refining privacy policies for human performance, facilitating scientific innovation, shaping e-governance, and reinforcing public confidence using nanotechnology. Human body is a thermodynamic heat engine in providing effective mechanical work as a function of psyche, conventional fuel transformation into enriched protein meal, and balancing of work-life fulcrum. The triboelectric effect of rubbing surfaces, interfaces, and interphases is invincible from the large field of the planet to nanodomains.

## 1 Introduction

**Fig. 1:**
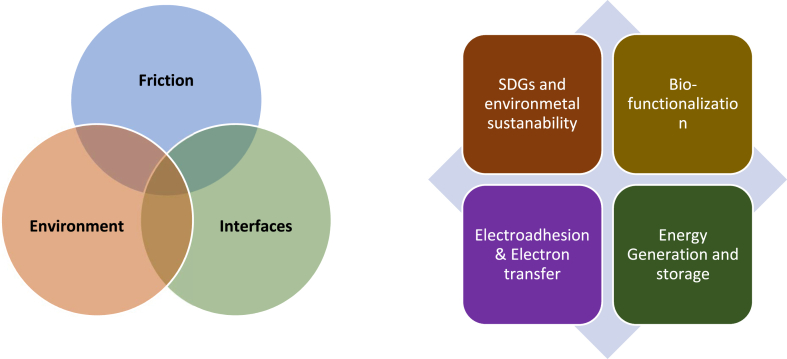
The nanoscience and nanotechnology in a diverse engineering application such as environmental sustainability in rationalization of environmental loadings, biofunctionalization of biosensors, electroadhesion due to inherent presence of supramolecular affinity of heterogeneous surfaces, and energy generation.

The importance of climate action including COVID-19 virus and antibody testing for preventing the spread of infectious diseases for herd immunity is the forefront of sustainability of good health and well-beings [[1], [2], [3], [4], [5], [6], [7], [8]]. The electromagnetic force is one of the four fundamental forces of Nature due to the interaction of charged molecules or electroadhesion in providing *in situ* rubbing at mechanical contacts. Mimicry of planetary electromagnetism is a fundamental principle of devices operations such as electric motors, solenoids, transformers, inductors, and generators preferred conventionally during energy transformation. The miniaturization of matter to atomic scale for resolving engineering applications have paved the foundation of nanoscience and nanotechnology by Feynman for delivering a diverse domain of physics lecture six decades ago [9]. The ability to design biological macromolecules had secured a pathway for innovation from microtechnology to nanotechnology for characterization, repair, and manipulation of biological materials [[10], [11], [12]]. The nanoscience ahead of physicist Richard Feynman lecture (1959) and the nanotechnology in molecular manufacturing by Drexler (1986) have advances the opportunities for technological developments across the range of nanomedicine, life science, materials science, and electroadhesion [[13], [14], [15]]. The nanomaterials diverse applications are rendered in controlling the molecular size at the nanoscale for transformation of the physiochemical, optical, electronic, and mechanical properties [[16], [17], [18]]. Nanoscience and nanotechnology have been scientifically visible for the last few decades in biosensing, energy storage, and generation, cytotoxicity, and photonics due to excellent mechanochemical and surface functionalities (Fig. 1).

A few layers of nanoparticle (2–10 atomic thick) or less than 100 nm could erode the body's immune system from health consequences such as metal oxides/carbon nanotubes/quartz dust/asbestos particles from the evolution of reactive surfaces, particle toxicity, and damaging human cells [19]. The *in-situ* presence of cytotoxic nanomaterials in urban cities environment predicted from fuel combustion of petroleum products, reducing environmental cytotoxicity from amalgamation of hybrid and electric vehicles for energy storage by LIBs, and harmony of net mechanical efficiency of human thermodynamics have been virtually incorporated for promotion of SDGs/Paris Agreement/IPCC/FAME [[20], [21], [22], [23]]. Socioeconomy and the industrial revolution of the 21st century have envisioned nanoscience from the biomechanical domain to the triboelectric effect as a function of state variables. The nanoelectromechanical system's evolution from microelectromechanical systems is viable for an understanding of engineered molecular functionalities for diverse applications. Materials science for renewable energy conversion and storage, chemical catalysis and synthesis, optoelectronics, and environmental pollution treatment for sustainability will pave the foundations to solve the energy crisis through the advancement of green technology, atmospheric CO_2_ transformation into cheaper materials, and reducing greenhouse emissions [24]. The fourth-generation industrial revolution, Sustainable Development Goals, and geographical socioeconomic indicators have advanced in parallel for the achievement of scientific innovation from nanotechnology [25]. The biomedical demand for faster and reliable testing methodologies evidenced by political requirement has highlighted the advancements in nanotechnologies beyond applications of bio-sensing technology in healthcare, energy generation, chemical industry, and food security.

## 2 Carbon NPs

Quantum technology is perplexing during 21st century to spur various innovative breakthroughs from powerful digital devices useful for designing and development new drugs, functional nanomaterials, smaller and more precise sensors, secured information and communication technology, regulation of financial markets, and prediction of climate action of weathering than conventional technology. Carbon is an element of so far study in proposing the buckminsterfullerene hollow-cage structure of the C_60_ cluster revealing light on physiochemical properties of functional carbon nanoparticles particularly vital implications from evolution in combustion as well as of polyaromatic compounds [26]. The synthesis and preparation of needle like tubes molecular carbon structures ranging from a few to a few tens of nanometres in diameter is an engineering milestone of 1-D carbon nanomaterials or Carbon nanotubes (CNTs) in applications [27,28]. The electric field effect of atomic thick sheet of carbon is unstable at ambient conditions for embarking of oxidation or functional groups due to the presence of nature reactions [29]. The sp^2^ hybridization of carbon atoms ease superb physiochemical, thermal, electronic, optical properties for advancement of nanotechnology and promotion of sustainability [[30], [31], [32], [33], [34], [35], [36]]. A monoatomic thick hexagonally arranged carbon of graphene has created tremendous academic cum scientific interests in energy generation in aligned with a large specific surface area, high charge carrier mobility, excellent thermal conductivity, remarkable optical transparency, and superior permeability useful for membrane applications (Table 1). The electron transfer performance of graphene nanomaterials with cell membrane is a supramolecular cell adhesion for modulation of surface energy.

**Table 1:** The physiochemical, optical, thermal, and electronic properties of carbon nanomaterials for rendering in engineering applications [[30], [31], [32], [33], [34], [35], [36]].

Expression	Properties
Extraordinary anisotropy and mechanical properties with Young's modulus ∼1.0 TPa due to the strong covalent bonding of carbon atoms for a single layer of graphene sheet	*Young's Modulus*
sp^2^ hybridized carbon atoms arranged in honeycomb 2-D lattice of Graphene with high surface area density ∼2630 m^2^/g for surface functionalization in engineering applications	*Specific surface area*
Single layer graphene provides electron mobility as high as ∼200,000 cm^2^V^−1^s^−1^ for electron density of ∼2 × 10^11^ cm^−2^ useful for electron transferring in engineering applications	*Electronic*
Graphene nanosheet with over 100-fold anisotropy of thermal properties of the in-plane/out-of-plane orientations due to covalent sp^2^ bonding between carbon atoms in providing thermal conductivity 3000–5000 Wm^−1^K^−1^ whereas out-of-plane thermal conductivity is reduced by weak van der Waals coupling	*Thermal*
Optical transparency of graphene ∼97.7 % is the physical property for allowing light to pass nanomaterials without being scattered referred to as pellucidity or diaphaneity	*Optical*

The single layer thick carbon structure of graphene is attractive scientifically due to ultra-high mechanical strength, electronic performance, thermal conductivities, impermeability to gases as well as other properties for numerous engineering applications [37]. Graphene research publications has risen since 2004 due to mechanical isolation of graphene substrate by “Geim & Novoselov” for outstanding electronic properties, production, functionalization, and future outlooks [38]. The advancement of nanoscience has influenced the physicochemical properties of carbon dots such as nano size (<10 nm), functional groups, biocompatibility, fluorescence, Bioimaging, Theranostics, and nontoxicity of carbon dots ahead of fullerene, nanotubes, and graphene [39,40]. Carbon dots (CDs) are nanomaterials among the carbon-based nanomaterials gained an incredible interest in nanotechnology and biomedical science due to exclusive attributes including electron transfer rate, fluorescence property, excellent biocompatibility, chemical stability, economy, and large specific surface area [[41], [42], [43]]. The large surface area and electromechanical performance of graphene inspired 2-D hybrid systems have proven functional properties for the emerging needs in electronics and optoelectronics such as printed, flexible and wearable electronic devices [44]. Graphene nanoribbons are quantum materials that secure synergistic quantum physical effects carbon NPs such as extraordinarily high tensile strength, thermal and electrical conductivity, in addition to quantum effects by evolving 2-D nanostructure functionalities of ribbon-like shape to a spectrum of controllable quantum effects [45,46]. The diversity of random and engineered carbon particles has strong supramolecular interactions to biomechanical substrates due to electroadhesion, large surface area per unit mass, and presence of functional groups in conscious biosphere.

## 3 Environmental reactions & biofunctionalization

Human body is a thermodynamic heat engine based on 2nd law of thermodynamics in pursual of mechanical work cumulatively by man and machine as per the integrity of socioeconomic factors [[47], [48], [49], [50]]. CO_2_ absorption, utilization, and storage may reduce environmental loadings evolve from fossil fuel-based IC engines over urban cities useful for promotion of green technology at the forefront of pandemic [51,52]. The covid#19 outbreak is global challenge for mitigation of health systems in involvement of graphene-based nanotechnology such as biosensors, antimicrobial applications, and antiviral efficacy of high versatile carbon-based nanomaterials [53,54]. Carbon nanotubes (CNTs), graphene, and graphene oxide (GO), fullerene have been preferred in environmental monitoring, healthcare, and food safety control due to physiochemical characteristics such as ultra-mechanical strength, high surface area, functionalities, and versatility [55]. The interaction of cell membrane models with GO is expressed for potential harmfulness in a wide range of biologically membrane parameters such as cholesterol content, size, and charge to evaluate the outcome of interactions from the loss of membrane integrity with electrostatic attraction and inversely correlated with cholesterol content [56]. The studies of CNT genotoxicity indicated that implicit interaction of functional groups with genetic materials supported the concept that CNT is genotoxic via a pathway of oxidative DNA attack evolved from free radicals during CNT elicited inflammation [57,58]. Biofunctionalization of nanomaterials integrates performance parameters at the interface of biological with nanoparticles in providing a functional engineered platform (Table 2) for the recognition of the sensing ability. Graphene based nanomaterials (GBN) such as 0D fullerene, 1D carbon nanotubes, 2D Graphene/graphene oxides (GO)/few layers of graphene (FLG), and 3D graphite provide novel electron transfer phenomenon for scientific usefulness in many engineering applications up to biofunctionalization.

**Table 2:** The biofunctionalization of graphene family nanomaterials for biological applications such as biosensor development by using graphene or graphene-based nanomaterials, and the fundamental investigation of graphene-based nanomaterials for living cell studies included in tabular form [[53], [54], [55], [56], [57], [58]].

Synergy of graphene family nanomaterials biofunctionalization	Keywords
The global pandemic for detection and diagnosis of Covid#19 viruses for the knowledge of graphene-based nanotechnology for virus interaction as well as capturing and deliver antiviral drugs delivery	*Covid#19*
Graphene family nanomaterials evolve an inflammatory response by integrity of toll-like receptors (TLRs), activating the transcription factor NF-κB for signalling pathway in biological unit cells, regulation of immune functions, and reactive oxygen species (ROS) generation	*Cytotoxicity*
Nowadays biosensors are ubiquitous in sensing of biomedical diseases as well as a wide range of healthcare monitoring, disease progression, drug discovery, environmental monitoring, food control, and biomedical research	*Biosensors*
Carbon nanomaterials or graphene family members provide unique biofunctionalization in a wide range of applications in adsorbing DNA and adsorbed fluorophores for providing information	*DNA*
Food and beverages quality measures for the good health and well-being for monitoring by Graphene family nanomaterial in maintaining the hygiene of food and beverages as fuel oxidation of intake affected by qualitative parameters	*Food industry*

Nanoscience is providing ultra-functionalities such as electromechanical properties, biofunctionalization, large specific surface area, and strong supramolecular interactions of functional groups at biological with nanomaterials interface. The CNT nanomaterials have attracted a huge potential due to their physiochemistry and electromechanical performance at the nanoscale in curing new insights of applied perspectives [59]. Lung fibrous surface of human breathing system is the first line of supramolecular adhesion between inhaled carbon nanomaterials and the biological organism in assessment of pulmonary health effects [60]. Nanoscience and nanotechnology are a fast-emerging biomechanical domain preferred in drug delivery, tissue engineering, biosensing, SARS-CoV-2 low-cost monitoring/diagnosis, and cytotoxicity for functionalization at biological membrane with mechanical substrates [[61], [62], [63], [64], [65], [66]]. The human body defence mechanism treats nanoparticles as microorganisms to form fibres with a high proportion reactive surfaces alter the nanoparticles toxicity, severity, and harm human cells. Reverse engineering, backward engineering, and back engineering may provide deductive reasoning of SARS-CoV-2 pandemic reactions over mankind from nanoscience or nanotechnology. The pandemic in India is an environmental reaction of anthropogenic urban biosphere domain for a message of materials and energy modulation. The reaction forces of random carbon matter suspended with environment of urban umbrella is assessed from cytotoxicity of few layers of graphene oxide damaging biological membrane across respiratory system through oral tribology. The air borne syndrome of reactive environment may be reversible for transformation of energy expenditure, plant-based adsorption over sink zone of thermodynamic cycle, and enhancement of net mechanical efficiency in a local clean environment, and high protein proportional fuel intake for oral tribology and food processing.

## 4 Tribology & electroadhesion

Energy dissipation at tribology interface is ubiquitous in daily human life from Nano-, Micro, Meso-, Macro-, and large field of biosphere to harvest mechanical energy into electrical energy. From boundary lubrication or nanotribology to microelectromechanical systems in moving parts in presence of interfacial environment has proven to be a tribological strategy for controlling frictional drag of an engineered system [[67], [68], [69], [70], [71]]. The energy harvesting and reduction of environmental load can be made from transformation or researching of human motions, industrial modulation, hybrid mechanical machines, and residual mechanisms of piezo-, pyro-, thermo-, and triboelectric effect [72]. Frictional forces create during motion of body in motion regulating towards a rational manner for gaining leverage over mechanical energy losses for explaining nanoscale friction by means of interfacial environment of electrochemistry [73,74]. The triboelectric effect is a well-known phenomenon of nature in our lives at any time and any place from skin rubbing with fabrics, nanomaterials triboelectrification, and at residual surfaces [[75], [76], [77]]. An eco-friendly and naturally abundant bacterial nanocellulose based bio-triboelectric nanogenerator having unique functionalities such as biocompatibility, transparency, and reliability in resolving need of sustainable energy resources [78]. Cellulose nanofibrils are used for attaching nitro/methyl groups to cellulose molecules to change the tribopolarities for enhancing the triboelectric outputs; the nitro cellulose nanofibrils possesses a negative surface charge density of 85.8 μC m^−2^; the methyl cellulose nanofibrils possesses a positive surface charge density of 62.5 μC m^−2^, touching 71% and 52% of fluorinated ethylene propylene [79]. The mechanism and mechanics of triboelectrification is briefly and fundamentally outlined at the epidermis structure of hair and skin, impact of chemistry and environment, and the applications of the human skin triboelectric effect [80,81]. The rubbing of engineered surfaces evolves triboelectric effect viable for miniaturization of energy storage and generation due to the involvement of friction from nanodomain to large field of planet.

The ultrahigh surface charge density of 1090 μC m^−2^ is obtained using ultrathin dielectric layer in high vacuum or charge pumping techniques of atmosphere through triboelectrification effect [82]. The contact electrification and electrostatic induction is an emerging harvesting technology in reaching current area power density 313 W/m^2^ and their volume energy density 490 kW/m^3^ of output performance [83]. The triboelectric nanogenerators e-skin is fabricated by push–pull ionic electrets for the charge mobility through an ion-hopping mechanism for the generation of a higher output voltage and currents at electronegative PTFE interface for flexible, durable, commercially viable, and a higher electrical charge outputs at applied frequencies [84]. The electrical characteristics of graphene interface of covalent bonds, adsorption, π–π bonds, and lattice incorporation are governed by mechanisms; conversion of carbon's hybridized state; dipole-dipole interactions; orbital hybridization with an interface to understand the effect of their functionalization [85]. The smart electric devices, intelligent energy generation and storage that can interact to external stimuli may provide socioeconomic values such as graphene potential to revolutionize biomedicine, energy storage, and biosensors owing to its excellent electronic and mechanical properties [86]. The monolayer graphene single microelectrodes evolving electric charge of continuous aqueous flow provides an effective flow sensing technology in delivering performance metrics relatively higher than other conventional electrical scientific routes [87]. Self-powered engineered systems can perform without an external power supply for sensing, detection, data processing and data transmission such as nanogenerators based on piezoelectric effect and triboelectrification effect for transforming mechanical energy into electricity with utility in IoTs, medical science, and environmental monitoring [[88], [89], [90], [91]]. The breakthroughs in graphene based smart energy generation and storage can safeguard academic or scientific utility in the incoming decades.

## 5 Conclusions

The cytotoxicity evolved from environmental reactions of fossil fuel based mechanical machines is a materials energy imbalance of science policy society interface. The biosphere is conscious for creation of environmental reactions at the meeting domain of mankind with machines in pursual of socioeconomic forefront with respect to time. The role of nanotechnology for surface functionalization for energy generation and utilization, global health, the benefits in terms of improving the outcomes of infectious diseases, and controlling the proliferation including the possibility of designing integrated devices for biomedical applications enumerated at materials and energy interface:

• The scientific development of efficient and drug releasing platforms, the engineering of vaccines of controlled physiochemical properties, and the immune boosting response against environmental third bodies or pathogens have been envisioned by nanoscience during 21st century at the forefront of global coronavirus pandemic;
• Graphene based nanomaterials have excellent physiochemical and electroadhesion functionalization at biological membrane/supramolecular interactions of –NH_2_, –COOH, –OH functional groups for evolving surface tension;
• Graphene family nanomaterials have surface functional physiochemical properties viable in biosensing, electronics, energy storage and generation, and biomechanical applications of cytotoxicity;
• Friction, lubrication, and wear at interacting surfaces is useful for assessment of triboelectric effect of electromagnetism for electron transfer efficacy as a function of materials properties, surface science, distancing, environment, and rubbing speeds;
• Friction regulation, human behaviour, synchronization of global policies, modulation of conventional IC engines into hybrid and electric cars, and reserving green sink zone cumulatively absorb environmental load for synergistic health or well-beings;
• Environmental reactions from fossil fuel-based fuel in urban cities can lead to a wide range of diseases and health outcomes such as epigenetic modifications, DNA methylation, and noncoding RNAs;
• The health effects of environmental pollutants are urgently needed to identify and elucidate their adverse effects on human health;
• The identification of epigenetic biomarkers for exposure, adverse health effects caused by environmental pollutants, and exploring underlying mechanisms can be eased by nanotechnology;

Tribology at rubbing contacts of electromechanical domain is predominant during 21st century in India urban cities such as android phone, electronic devices, and touch pad for effective rationalization of tactile friction. The omnipresence of electromagnetic field over planet surface have created numerous tribology applications such as energy generation, fabric selection for human skin protection, and biofunctionalization.

## Ethics declaration

None experiments on living being for expressing a heterogeneous system.

## Funding resource

None funding in writing of paper.

## Data availability statement

Data is reinforced in the article expressed from secondary source included with references.

## CRediT authorship contribution statement

**Pankaj Tomar:** Writing – review & editing.

## Declaration of competing interest

None conflict of interests to declare.
